# A Near-Infrared Spectrometer Based on Novel Grating Light Modulators

**DOI:** 10.3390/s90403109

**Published:** 2009-04-24

**Authors:** Wei Wei, Shanglian Huang, Ning Wang, Zhu Jin, Jie Zhang, Weimin Chen

**Affiliations:** 1 Key Lab of Opto-electronic Technology and Systems, Education Ministry of China, Chongqing, 400044, P.R. China; E-Mails: slhuang@cqu.edu.cn(S.H.); wn1983@163.com(N.W.); jinzhu_0323@sina.com(Z.J.); zhangjie@cqu.edu.cn(J.Z.); wmchen@cqu.edu.cn(W.C.); 2 Optoelectronic Engineering Department, Chongqing University, Chongqing, 400044, P.R. China; 3 Microsystem Center, Chongqing University, Chongqing, 400044, P.R. China

**Keywords:** MOEMS, Near-infrared, Spectrometer, Grating light modulator

## Abstract

A near-infrared spectrometer based on novel MOEMS grating light modulators is proposed. The spectrum detection method that combines a grating light modulator array with a single near-infrared detector has been applied. Firstly, optics theory has been used to analyze the essential principles of the proposed spectroscopic sensor. Secondly, the grating light modulators have been designed and fabricated by micro-machining technology. Finally, the principles of this spectroscopic sensor have been validated and its key parameters have been tested by experiments. The result shows that the spectral resolution is better than 10 nm, the wavelength deviation is less than 1 nm, the deviation of the intensity of peak wavelength is no more than 0.5%, the driving voltage of grating light modulators array device is below 25 V and the response frequency of it is about 5 kHz. With low cost, satisfactory precision, portability and other advantages, the spectrometer should find potential applications in food safety and quality monitoring, pharmaceutical identification and agriculture product quality classification.

## 1. Introduction

Near-infrared spectroscopy [1–3] is widely used for chemical analysis, food safety and quality monitoring, materials inspection and the monitoring of dynamic process, etc. Most established and classical methods in this field can be grouped into two classes: (1) Dispersive methods, including scanned-grating monochromators or optical multichannel analyzers (OMA) typically using a detector array. (2) Nondispersive methods, including arrays or sequences of fixed filters, or Fourier Transform spectroscopy (FTIR). Each of these techniques provides different combinations of resolution, speed, sensitivity and cost.

Micro-opto-electromechanical systems (MOEMS) technology has experienced a rapid progress in recent decades. A near-infrared spectrometer based on this technology with many advantages such as cost effectiveness, portability, low power consumption, high speed, and miniaturization has become one of the most interesting research topics in the near-infrared spectroscopy field. In particular, this kind of handheld spectroscopic sensor with a competitive price can be used for a range of applications that have been restricted to larger and more expensive instruments up to now. Kraft has proposed a single-detector micro-electro-mechanical scanning grating spectrometer [4]. The spectral scanning can be accomplished by the rotation of an oscillating reflection grating, which can avoid using an expensive near-infrared detector array. Geller has proposed the digital transform spectrometer (DTS) [5–6] based on electrically programmable diffractive MOEMS chip. The diffractive MOEMS chip is a time encoding mask and the intensity of each wavelength band can be acquired by changing the diffraction model of pixels. The full spectrum can be obtained using a single near-infrared detector in combination with the MOEMS chip. In addition to the major cost advantage, the dark current, detector noise, and thermal drift in the detectivity is common to all wavelengths, eliminating detector-element variability as a potential limitation on performance.

Since many exacerbated problems in developing countries such as unsafe food and fake medicines present potential human health hazards, there is a great and urgent demand for a spectroscopic sensor with good enough precision, high reliability and very low cost. Furthermore, due to their fabrication complexity, the price of both the above mentioned spectrometers are not low enough to be acceptable for worldwide applications, let alone for most developing counties. Therefore, in this paper a near-infrared spectrometer based on the novel MOEMS grating light modulator (GLM) [7–12] device is proposed. Compared with other MOEMS near-infrared spectrometers, the fabrication of the MOEMS chip in our spectroscopic senor is easier, allowing for lower cost in applications such as primary food quality monitoring, pharmaceutical identification and agriculture product quality classification. The major goal of this paper is to analyze the basic principles of the spectrum sensing system and show our related experimental results.

## 2. Optical system layout

**Figure 1.**
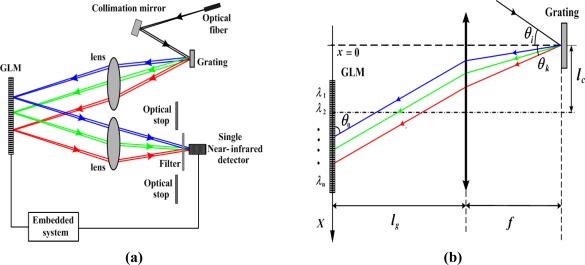
(a) Architecture of spectrometer (b) Position relationship among GLM, lens and fixed grating.

The architecture of the near-infrared spectrometer is shown in Figure 1(a). The light is dispersed spatially with a fixed diffraction grating and then directed onto the diffractive MOEMS chip, which contains an array of grating light modulators consisting of *N* pixels. The MOEMS chip in combination with the fixed diffraction grating constitutes a series of electrically programmable optical reflectors, which select one spectral region at a time, diffracting all other wavelengths to other directions. All the reflected light at one selected spectral region is collected onto a single photo-detector and the diffraction light is blocked by the optical stop. By scanning each pixel across the MOEMS chip, the optical energy of each wavelength band can be acquired in sequence through a scanning period, so that a full spectrum can be obtained instantaneously. To avoid the influence of the second-order diffraction visible light, a near-infrared long-pass filter is usually placed in front of the detector.

As shown in Figure 1(b), the fixed diffraction grating is placed on the focal plane of the Fourier lens and all the dispersive light incident on the grating light modulator array is at an angle of *θ_0_*. The relationship between the wavelength of the diffracted light and the position of pixels can be derived as:

(1) $$\lambda_{n}=d[\text{sin}({\text{tan}}^{-1}(x_{n}-l_{g}\;\text{tan}\;\theta_{0}/f))-\text{sin}\;\theta_{i}]$$

where *λ_n_* is the central wavelength of the diffraction light shooting on the *n^th^* pixel, *d* is the grating constant of the fixed grating, and *x_n_* is the central position of the *n^th^* pixel on X-axis, which is determined by the design size of the grating light modulator. Formula (1) is fundamental for designing the optical system, which determines the spectral range, resolution, and the dimensions of the GLM.

## 3. The spectra detection principle based on grating light modulators

### 3.1. The optical principle of grating light modulator

**Figure 2.**
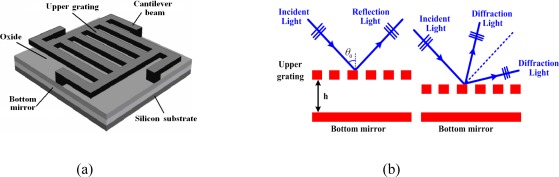
(a) Structure of a single GLM. (b) Optical model of GLM.

A single GLM consists of the upper moveable grating, the silicon dioxide layer, the bottom mirror, and the substrate. The upper moveable grating and bottom mirror, made up of the aluminum, compose a phase grating. Figure 2(a) illustrates the structure of a single GLM. When voltage actuated, the GLM becomes a tunable phase grating device and the optical model is shown in Figure 2(b).

Fourier Optics theory is used to explain the optical principle of a single GLM pixel. The spatially dispersed light shooting on the GLM array in the angle of *θ_0_*, is illuminated in Figure 1(b). The illuminating function of each GLM pixel is as follows:

(2) $$\text{exp}(x,y)=\text{exp}(j2\pi xf_{0})$$

where *f_0_*=*sinθ_0_*/*λ*. And the transmittance function of each GLM pixel *t_s_* can be expressed as:

(3) $$t_{s}\;(x,y)=(\sum_{m=-\infty }^{\infty }\mathrm{rect}(\frac{x+{\mathrm{md}}^{\prime }}{a})+e^{\frac{j4\pi h}{\lambda \;\text{cos}\;\theta_{0}}}\sum_{m=-\infty }^{\infty }\mathrm{rect}(\frac{x+{\mathrm{md}}^{\prime }+d^{\prime }/2}{a}))\mathrm{rect}(\frac{x}{W})\mathrm{rect}(\frac{y}{L})$$

where *a* is the width of upper grating ribbon, m is an integer, *d'* is the grating constant of GLM, *L* and *W* are the length and width of the GLM pixel respectively, *h* is the distance between upper moveable grating and the bottom mirror, and 4hπ/λcosθ_0_ equals to the phase difference between the light reflected from the upper grating and that from the bottom mirror.

The diffraction pattern [13] is seen to be as:

(4) $$U_{s}\;(x^{\prime },y^{\prime })=\frac{1}{j\lambda z}\;\text{exp}(\mathrm{jkz})\text{exp}[j\frac{k}{2z}({x^{\prime }}^{2}+{y^{\prime }}^{2})]\tilde{F}\{e(x,y)t_{s}\;(x,y)\}$$

where *k*(equals *2π/λ*) is the wavenumber, *λ* is the wavelength of the incident light, *f_x_=x/λz, f_y_=y/λz*, and *F̃* represents the Fourier transform operation. Intensity distribution of the diffraction light can be calculated by formula (4):

(5) $$I_{s}\;(x^{\prime },y^{\prime })=|U_{s}\;(x^{\prime },y^{\prime }){|}^{2}=|\frac{A}{\lambda z}T_{s}(\frac{x}{\lambda z},\frac{y}{\lambda z}){|}^{2}$$

When considering the spectra bandwidth *Δλ* of the incident light onto each pixel, the intensity of the reflected light onto the detector then can be derived as:

(6) $$\begin{array}{ll} I(x^{\prime },y^{\prime })= & \int_{\lambda_{n}-\Delta \lambda_{n}/2}^{\lambda_{n}+\Delta \lambda_{n}/2}{\left(\frac{\mathrm{aLW}}{d^{\prime }}\right)}^{2}\text{sin}\;c^{2}(Lf_{y})\sum_{m=-\infty }^{\infty }\text{sin}\;c^{2}(\frac{\mathrm{am}}{d^{\prime }})\;\text{sin}\;c^{2}(W(f_{x}-f_{0}-m/d^{\prime }))\\ & \times (1+e^{j(m\pi +\frac{4h\pi }{\lambda \;\text{cos}\;\theta_{0}})})d\lambda \end{array}$$

**Figure 3.**
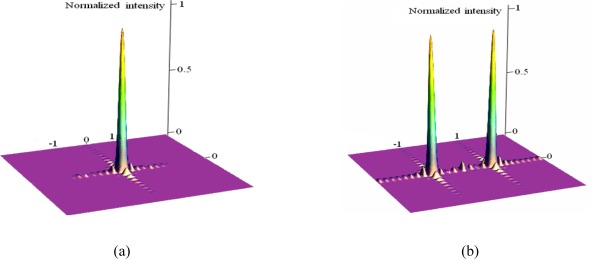
(a) Intensity of the zero order, when phase difference is 2*m*π. (b) Intensity of the zero order, when phase difference is (2*m*+1)π.

Figure 3 illustrates the diffraction pattern of the single grating light modulator calculated by formula (6), where *a*=5 μm, *d'*=10 μm, *L*=50 μm, *W*=50 μm, *λ_n_*=1,300 nm, *Δλ_n_*=6.25 nm.

Figure 3(a) is the calculated intensity on the detector when the phase difference of GLM is 2*m*π, while Figure 3(b) is the intensity when the phase difference of GLM is (2*m*+1) π. It can be seen that when voltage V_on_ actuated, the energy of the zero order, almost equaling that of the incident light, reaches maximum, the phase difference is 2*m*π and the pixel is *on* and when a voltage V_off_ is actuated, the energy reaches nearly zero, when the phase difference is (2*m*+1) π and the pixel is *off*. Suppose the reflection efficiency is *T*, which is the ratio between the intensity of the zero order and the incident light, when the pixel is *on*, while the reflected ratio is *T_0_*, when the pixel is *off*. The calculation results derived from formula (6) shows that *T* and *T_0_* equals 0.94 and 0.008 respectively. Apparently, the grating light modulator acts as a programmable pixilated optical switch with high contrast.

### 3.2. The sampling of spectra based on GLM array

The GLM array acts as a series of pixilated optical switches in the spectrometer. What is different from large-scale spectrometer is that in handheld MOEMS spectrometer, miniature dimension of optical system, simple instrument structure and cost effectiveness are required. Therefore, the goal of optical design is mainly to ensure there is good spectral resolution in X axis (the dispersive direction), without considering so much about the optical aberration in Y axis. The size of the light spot on a GLM pixel in Y axis is approximately from 500 μm to 800 μm, which is tens of times than that in X axis, and in our sensor, the size of a single GLM is 52 μm×52 μm. Consequently, to better make use of the optical energy in Y axis, 16 grating light modulators in Y direction are used as one pixel column. In other words, a pixel in spectrometer is not a single grating light modulator but a column of them.

**Figure 4.**
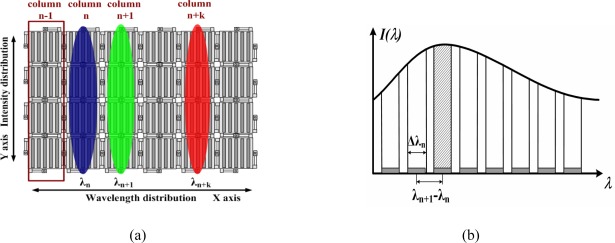
(a) Diffracted light incident on a GLM array chip. (b) Spectrum sampling by GLM array.

The spectrum can be obtained by a string-line pattern scanning along the X-axis direction. The diffracted light shooting on GLM array is shown in Figure 4(a). Essentially, the GLM array is used as a linear array in the spectrometer. Consequently there is no crosstalk [14–15] in its driving circuit, so that will lead to better optical switch performance. The sampling process by GLM array illustrated in Figure 4(b) shows that each pixel can only control a specifically corresponding piece of the incident wavelength band. Both the single near-infrared detector and the GLM array are controlled by a digital signal processor simultaneously. The sampling process can be described as that each pixel is turned on in sequence.

The discrete spectrum by sampling can be derived as:

(7) $$\begin{array}{ll} I_{s}\;(\lambda )=I(\lambda_{n})= & T\times \int_{-\infty }^{+\infty }I(\lambda )\mathrm{rect}(\frac{\lambda -\lambda_{n}}{\Delta \lambda_{n}})d\lambda +\\ & T_{0}\times [\sum_{i=1}^{n-1}\int_{-\infty }^{+\infty }I(\lambda )\mathrm{rect}(\frac{\lambda -\lambda_{i}}{\Delta \lambda_{i}})d\lambda +\sum_{i=n+1}^{N}\int_{-\infty }^{+\infty }I(\lambda )\mathrm{rect}(\frac{\lambda -\lambda_{i}}{\Delta \lambda_{i}})d\lambda ] \end{array}$$

where *N* is the number of pixels, *Δλ_i_* is the spectra bandwidth of the incident light on the *i^th^* pixel.

### 3.3. The instrument spectra reconstruction

The instrument spectra could be reconstructed using the discrete spectrum obtained by GLM sampling, according to Nyquist–Shannon sampling theorem [16]. As long as the sampling instants are sufficiently close, the spectral signal can be reconstructed accurately. By using a low pass filter, the precise interpolation [16] can be carried out between the sample points. The interpretation of the reconstruction of *I*(*λ*) as a process of interpolation becomes evident when we consider the effect in the spectrum signal domain of the low pass filter. The reconstructed instrument spectra can be derived as:

(8) $$\begin{array}{ll} \hat{I}(\lambda ) & =I_{s}(\lambda )⊗r(\lambda )\\ & =\{T\times \int_{-\infty }^{+\infty }I(\lambda )\mathrm{rect}(\frac{\lambda -\lambda_{n}}{\Delta \lambda_{n}})d\lambda +T_{0}\times [\sum_{i=1}^{n-1}\int_{-\infty }^{+\infty }I(\lambda )\mathrm{rect}(\frac{\lambda -\lambda_{i}}{\Delta \lambda_{i}})d\lambda \\ & +\sum_{i=n+1}^{N}\int_{-\infty }^{+\infty }I(\lambda )\mathrm{rect}(\frac{\lambda -\lambda_{i}}{\Delta \lambda_{i}})d\lambda ]\}⊗\frac{\text{sin}\;c(\lambda \Omega_{c})}{\lambda \pi } \end{array}$$

where *r*(*λ*) is an ideal low pass filter, *Ω_c_* is the cutoff frequency of the low pass filter.

**Figure 5.**
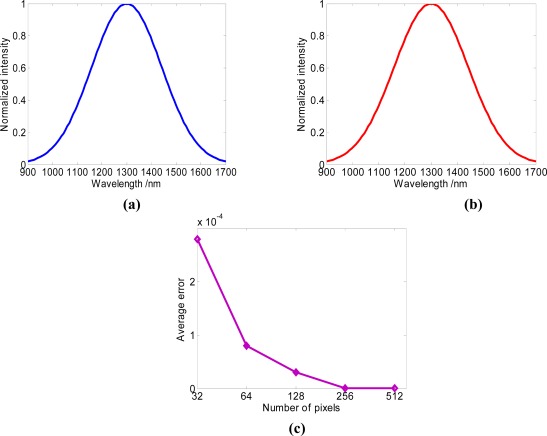
(a) Original spectrum (b) Reconstructed spectrum by 512 pixels (c) Relationship between average error and the number of pixels.

The spectra detection process was simulated with Matlab. The original spectrum is created by a Gaussian function (peak wavelength is 1,300 nm and the square error is 40,000) as shown in Figure 5(a). Choose the initial parameters as follows: the spectra range is from 900 nm to 1,700 nm, *N*=128, *θ_i_*=45°, *f*=50 mm, *lg*=52 mm, the *λ_n_* and *Δλ_n_* can be acquired from formula (1). Then, according to formula (8), choosing a cubic spline interpolation [17], the reconstructed spectrum can be acquired, shown in Figure 5(b). The average error *e*[*I*(*n*)] can be acquired by calculating the average error of the intensity of each pixel between the original spectrum and the reconstructed spectrum. What can be seen from Figure 5(c) is that the average error is tiny and becomes smaller with the number of pixels increasing. The diffraction ratio *T* and *T_0_* of the modulator do not influence the precision of reconstructed spectrum. When the number of pixels is 64, the average error is less than 0.0001. Consequently, the full spectrum can be reconstructed accurately by this method.

## 4. Experiments

### 4.1. The design and fabrication of GLM

To match the size of a diffracted light spot in the miniature spectrometer, the Y axis size of a single GLM should not be too small [18]. However, for a single GLM, the larger the size in the Y axis, the more difficult fabrication would be. Moreover, the difficulty increases exponentially. If we design a GLM of 50 μm × 500 μm size, that is, the size of which in X-axis is 50 μm and that in Y-axis is 500 μm, the stress and temperature during micro processing will cause a worse flatness of the upper movable grating, that would directly lead to a large decrease in the optical switch performance, and even lead the upper movable grating and lower mirror adhere together. Therefore, one effective way is required to make the flatness of upper grating less than *λ/10* (*λ* is the wavelength of incident light) for diffraction elements. For instance, the programmable grating used in digital transform spectrometer proposed by Polychromix, Inc. has a MOEMS grating designed with multi-layer structure to solve this problem. To decrease the cost and complexity of micro processing technology, meanwhile ensure high optical performance and enough optical energy for the spectroscopic sensor, several modulators in a column are used as one pixel and controlled simultaneously in our GLM array device. This new structure of GLM can not only reduce the difficulty of the processing and the operating voltage of the device, but also improve the response speed and flatness of the modulator.

The GLM is fabricated using surface micro-machining technology [19]. The stages of fabrication are as follows: (1) 500 nm–600 nm thick thermal silicon dioxide is deposited by furnace on a <100> p-type silicon wafer, for electrical isolation among bottom electrodes, (2) evaporation of aluminum of 100 nm, (3) lithography of aluminum to form bottom reflector surface and address electrodes, (4) 280 nm thick silicon dioxide dielectric layer was deposited as protection layer by plasma enhanced chemical vapor deposition (PECVD), (5) 600 nm thick aluminum is sputtered, (6) lithography of aluminum to form support posts, (7) polyimide is spin-coated as a sacrificial layer, (8) lithography of vias of posts, (9) evaporation of structural metal layer, (10) 100 nm thick aluminum is evaporated and then lithographed to form the upper moveable grating, (11) release of the sacrificial layer, (12) the grating light modulator has been fabricated.

**Figure 7.**
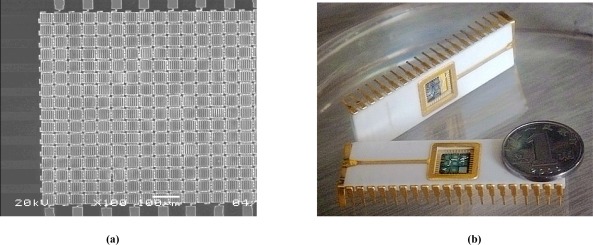
(a) The SEM photo of the fabricated GLM array (b) IC package of GLM array.

Figure 7(a) is the scanning electron microscopy photograph of the fabricated GLM array. The dimension of each single modulator is 52 μm × 52 μm and the width of the upper grating ribbon is 4 μm. Figure 7(b) shows the IC package of the grating light modulator array.

### 4.2. The experiments of spectra detection

To demonstrate the validity of the principle of this kind of spectrometer and to measure the basic characteristics of the spectrometer, an experiment was set up. The architecture of the experimental system is shown in Figure 1(a), above. The experimental device consisted of a tungsten halogen lamp (10 w, Avalight-HAL, Holland), a high pressure mercury lamp (200 w, GY-6, Tuopu Corporation, China), multi-mode optical fiber (100 μm in diameter, numerical aperture is 0.22), a rectangular calibration mirror (focal length equals 5 mm), a plane reflection grating (400 lines/mm), combined Fourier lens (focal length equals 40 mm), a GLM array device, optical stops, a low-noise current amplifier (Stanford model SR560, USA), an oscillograph (Wavepro 7300, USA), an InGaAs detector (the spectrum response range is from 900 nm to 1,700 nm) and a GLM array driving circuit.

According to the analysis above, the precision of the reconstructed spectrum is higher as the number of the pixels increases. Currently, we have fabricated the GLM chip with 32 sampling wavelength points. Through the calibration of three peak wavelength 1,357 nm, 1,367 nm and 1,395 nm which are the characteristic spectrum lines of the high pressure mercury lamp, the detection spectral range of our experimental system is from 1,320 nm to 1,400 nm.

#### 4.2.1. Spectral Resolution test

**Figure 8.**
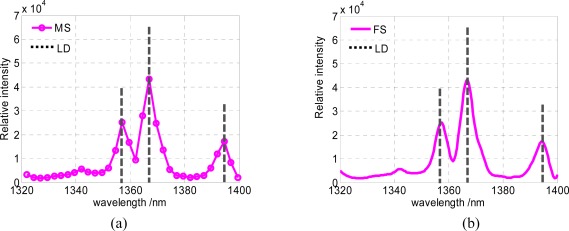
(a) Sampling spectrum of the high pressure mercury lamp. (b) Fitting spectrum curve of the high pressure mercury lamp.

To evaluate the effective spectral resolution of the spectrometer based on the GLM array, an experiment has been carried out to measure the emission spectrum of a standard high pressure mercury lamp. The results are shown in Figure 8, where MS is the measured spectrum, LD is the literature data and FS is the fitting spectrum. Figure 8(a) is the sampling spectrum by a GLM array device, Figure 8(b) is the fitting spectrum curve. The two characteristic peak wavelengths (1,357 nm and 1,367 nm) can obviously be distinguished. Therefore, the spectral resolution of this spectrum detection system is better than 10 nm. For most applications of a near-infrared spectrometer as a pocket-sized or handheld spectra analyzer, this resolution is in good enough level.

#### 4.2.2. Wavelength accuracy test

**Figure 9.**
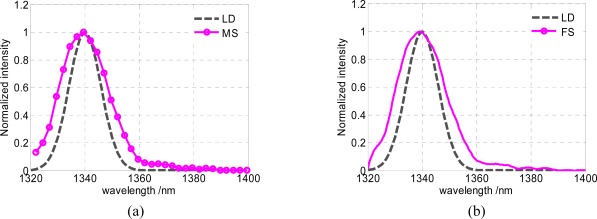
(a) Sampling spectrum of the 1,340 nm interference filter. (b) Fitting spectrum curve of the 1,340 nm interference filter.

The spectrum accuracy is demanded below ±1.5 nm for miniaturization near-infrared spectroscopic sensors, to ensure among which the effective transmissibility of the correct model. The transmittance spectrum of 1,340 nm narrow bandwidth interference filter illuminated by a tungsten halogen lamp has been tested 20 times. Spectral accuracy has been tested by comparing the measured spectra with the literature data. The average result can be seen from Figure 9. The full width at half maximum (FWHM) is 20 nm and the peak wavelength is 1,339.5 nm, hence the wavelength deviation of this spectrum detection system is less than 1 nm.

#### 4.2.3. System stability test

**Figure 10.**
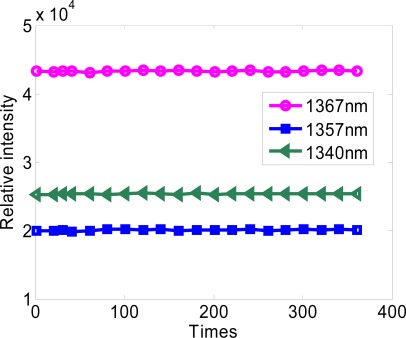
System stability test.

The stability of a spectrometer, also called repeatability and reproducibility, is related to the change of light intensity sampled by a pixel when the light source is determinate. The relative intensity of three peak wavelength aforementioned has been tested. Figure 10 shows the relationship between the relative intensity and the spectra scanning times. For the deviation of the intensity of peak wavelength 1,340 nm is about 0.4%, the deviation of the intensity of the peak wavelength 1,357 nm is about 0.3% and the deviation of the intensity of the peak wavelength 1,367 nm is about 0.3%. All the deviation of intensity of peak wavelengths is below 0.5%, so that this spectrum detection system is quite steady and reliable for a handheld instrument.

#### 4.2.4. Response characteristics test

**Figure 11.**
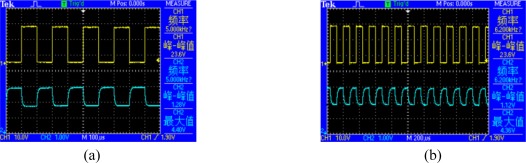
GLM response characteristics. (a) Response characteristics of the GLM actuated by a 5 KHz frequency square-wave. (b) Response characteristics of the GLM actuated by a 6.2 KHz frequency square-wave.

The response speed and the driving voltage of GLM are also important to the performance of the spectrometer, which determine the spectrum scanning speed and the miniaturization degree of this instrument. One of the pixels is actuated by a square-wave signal of different frequency generated by a signal generator, meanwhile, no voltage is actuated on other pixels. The single InGaAs detector is connected to a current amplifier and an oscillograph. The results shown in Figure 11 indicate that when the frequency of the actuated voltage is 5 KHz and the driving voltage is 23.6 V, the detected signal can remain undistorted, which can be seen in Figure 11(a) where that the upper signal is the actuating signal and the other one is the detected signal. Nevertheless, when the frequency of the actuated voltage is 6.2 KHz and the driving voltage is also 23.6 V, the detected signal is distorted and attenuated, which can be seen in Figure 11(b), where the upper signal is the actuating signal and the other is the detected signal. Therefore, the suitable response frequency is about 5 KHz and the driving voltage is no more than 25 V. Furthermore, if the total number of pixels is 512, theoretically it will take about 100 ms to acquire a full scan spectrum.

## 5. Conclusions and Outlook

A near-infrared spectrometer based on a novel grating light modulator is proposed in this paper. The basic principles of this device is analyzed in detail. First, the optical system is presented. The intensity of each wavelength band can be selected by GLM array device and detected by the single near-infrared detector in sequence. The process of spectrum detecting is analyzed and simulated. The result shows that this spectrometer can reconstruct the original spectrum precisely. If the number of pixels increases, the precision of the reconstructed spectrum becomes higher. Secondly, the GLM array device has been designed and fabricated by micro-machining technology. Finally, the principle of this kind of spectrometer combining the novel grating light modulators with a single near-infrared detector has been validated and the basic characteristics of the spectrometer have been tested. The results of experiments show that over the 1,320 nm to 1,400 nm spectral region, the spectral resolution is better than 10 nm, the wavelength deviation is less than 1 nm and the deviation of the peak wavelength intensity is no more than 0.5%.

With low cost, good enough precision, portability, high speed and other advantages, the spectrometer should have potential applications in the fields of food safety and quality monitoring, pharmaceutical identification, agriculture product classification and the monitoring of dynamic process. In addition, a huge sensing and monitoring network could be set up based on this kind of sensor, which would open a new application for near-infrared spectroscopic sensor technology. A near-infrared spectrometer with the detection spectral range from 900 nm to 1,700 nm based on our grating light modulators in now under development, and will be reported in future paper.

## Figures and Tables

**Figure 6.**
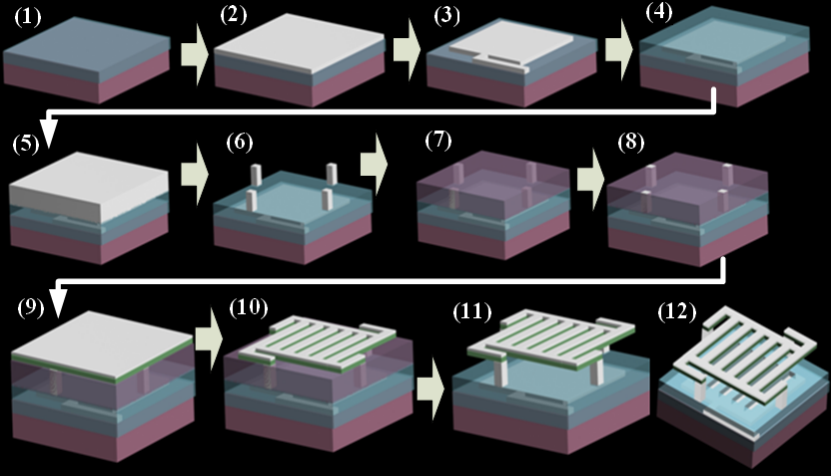
GLM fabrication flow.
